# Downregulation of TNIP1 Expression Leads to Increased Proliferation of Human Keratinocytes and Severer Psoriasis-Like Conditions in an Imiquimod-Induced Mouse Model of Dermatitis

**DOI:** 10.1371/journal.pone.0127957

**Published:** 2015-06-05

**Authors:** Yan Chen, Heng Yan, Zhiqiang Song, Fangru Chen, Huan Wang, Jun Niu, Xiaowei Shi, Dongmei Zhang, Na Zhang, Zhifang Zhai, Baiyu Zhong, Liangjin Cheng, Tian Qian, Fei Hao

**Affiliations:** Department of Dermatology, Southwest Hospital, Third Military Medical University, Chongqing, China; CNRS-University of Toulouse, FRANCE

## Abstract

Psoriasis is a chronic, inflammatory skin disease involving both environmental and genetic factors. According to genome-wide association studies (GWAS), the *TNIP1* gene, which encodes the TNF-α–induced protein 3-interacting protein 1 (TNIP1), is strongly linked to the susceptibility of psoriasis. TNIP1 is a widely expressed ubiquitin sensor that binds to the ubiquitin-editing protein A20 and restricts TNF- and TLR-induced signals. In our study, TNIP1 expression decreased in specimens of epidermis affected by psoriasis. Based on previous studies suggesting a role for TNIP1 in modulating cancer cell growth, we investigated its role in keratinocyte proliferation, which is clearly abnormal in psoriasis. To mimic the downregulation or upregulation of TNIP1 in HaCaT cells and primary human keratinocytes (PHKs), we used a *TNIP1* specific small interfering hairpin RNA (TNIP1 shRNA) lentiviral vector or a recombinant TNIP1 (rTNIP1) lentiviral vector, respectively. Blocking TNIP1 expression increased keratinocyte proliferation, while overexpression of TNIP1 decreased keratinocyte proliferation. Furthermore, we showed that TNIP1 signaling might involve extracellular signal-regulated kinase1/2 (Erk1/2) and CCAAT/enhancer-binding protein β (C/EBPβ) activity. Intradermal injection of TNIP1 shRNA in BALB/c mice led to exaggerated psoriatic conditions in imiquimod (IMQ)-induced psoriasis-like dermatitis. These findings indicate that TNIP1 has a protective role in psoriasis and therefore could be a promising therapeutic target.

## Introduction

Psoriasis is a common chronic inflammatory skin disorder affecting 1–2% of the northern American and European populations [1]. It has characteristic hitological changes, including epidermal hyperproliferation, infiltration of T cells and dendritic cells, and a distinct increase in skin angiogenesis. While the etiology is largely unclear, previous studies have shown that dermal injection of immune cells could induce psoriasis [2], and abrogation of activation protein 1 (AP1) pathway in keratinocyte signaling could lead to psoriasiform hyperplasia in mice [3]. Thus, both immunological and keratinocyte dysfunction are sufficient to initiate psoriasis-like skin disease. In addition, genetic components, as demonstrated by familial aggregation studies, are clearly involved [4]. At least 36 different loci have been identified as susceptibility loci of psoriasis by GWAS [5], including the *TNIP1* gene, which encodes TNF-α–induced protein 3-interacting protein 1 (TNIP1), as well as the tumor necrosis factor α-induced protein 3 (*TNFAIP3)* gene, which encodes protein A20 [6, 7]. Besides psoriasis, the *TNIP1* and *TNFAIP3* gene have been associated with systemic lupus erythematosus (SLE) [8, 9]. In fact, the CC genotype of rs10036748 in *TNIP1* is protective against SLE in European populations [9], as well as in Chinese Han population [9, 10]. Further study has shown that the G allele of rs610604 in the *TNFAIP3* gene correlates with a good response to TNF blockers in patients with psoriasis [11]. However, the mechanisms of how these susceptibility loci and their encoded proteins contribute to the pathogenesis of psoriasis remain largely unclear.

TNIP1, a widely expressed ubiquitin-binding protein [12], belongs to the TNIPs family and includes three different intracellular proteins, TNIP1, TNIP2 and TNIP3 [13]. TNIP1 interacts with the deubiquitylase A20 [14] and inhibits NF-κB transcriptional activity [15–18]. Psoriatic skin displayed a 1.47-fold increase in the mRNA level of *TNIP1*, when compared with uninvolved skin [6], suggesting that TNIP1 may have a role in the pathogenesis of psoriasis. Similar to TNIP1, A20 is also known to function as a negative regulator of NF-κB activity. Although TNIP1 interacts with A20 and shares a common role in repressing NF-κB activity [19, 20], TNIP1 can function independently of A20 [17, 21–23]. Interestingly, skin sections from A20-deficient mice revealed thickened epidermis without inflammation [19], suggesting a role for A20 in skin growth. However, whether TNIP1 shares the same role as A20 in skin growth remains unknown.

Despite the current focus on immune disorder, especially on innate immune cells in the pathogenesis of psoriasis [24–26], keratinocytes, the most predominant cell type in human epidermis, are characterized by hyperproliferation and aberrant terminal differentiation, resulting in the formation of plaque with a scaling surface in patients affected by psoriasis. Keratinocytes also have a profound influence on the immune system by producing different inflammatory cytokines and chemokines [27]. Since psoriasis is characterized by excessive epidermal growth, we postulated whether TNIP1 could affect the proliferation of keratinocytes. In fact, one study showed that overexpression of TNIP1 inhibited the growth of EGF receptor-overexpressing tumor cells [28]. Similarly, TNIP2, another member of the TNIP family, delays liver generation when overexpressed [29].

To date, the definite and precise role of TNIP1 in psoriatic epidermal cells remains unclear. The aim of this study was to determine the potential role of TNIP1 in psoriasis. Our data showed that TNIP1 played a role in modulating the proliferation of human keratinocytes and exaggerated IMQ-induced psoriasis-like dermatitis in mice. These findings may constitute an attractive target for therapeutic interventions for psoriasis.

## Materials and Methods

The experimental protocol was established according to the ethical guidelines of the Helsinki Declaration and was approved by the Human Ethics Committee of Southwest Hospital of the Third Military Medical University in Chongqing, China. Written informed consent was obtained from individual participants. Written informed consent was obtained from the guardians on behalf of the children enrolled in this study. All animal studies were approved by the animal ethics committee of the Third Military Medical University according to Dutch legislation on animal experiments. Surgery was performed under sodium pentobarbital anesthesia, and all efforts were made to minimize suffering.

### Cell cultures

HaCaT, a spontaneously immortalized human keratinocyte cell line [30], was purchased from Cell Resource Center (Beijing, China). Cells were cultured in RPMI 1640 (Hyclone, Logan, UT, USA) with 10% fetal bovine serum (FBS, Hyclone) under a humidified atmosphere containing 5% CO_2_ at 37°C. PHKs were isolated from normal foreskin discarded during circumcision of two patients (aged 9 years and 15 years, respectively) as previously described [31, 32]. Briefly, an epidermal cell suspension was obtained by incubating dispase-separated epidermal sheets in 0.25% trypsin solution at 37°C for 15 min. Cells were cultured on tissue culture plates coated with Collagen IV (Sigma, USA) in the presence of defined keratinocyte serum-free media (DK-SFM) (Gibco, Carlsbad, CA) at 37°C in humidified atmosphere with 5% CO_2_. Pan-Cytokeratin AE1/AE3 was positive after PHKs were exposed to 10% FBS for 24 h, as confirmed by immunofluorescence staining (S1 Fig). The experiments on PHKs were randomly conducted at passages 2–4.

### Immunofluorescence staining and confocal microscopy

HaCaT cells or PHKs were first fixed in acetone, permeabilized with 0.25% Triton for 10 min, blocked with 1% BSA for 30 min, and then incubated with mice anti-human TNIP1 monoclonal antibody (1:200 dilution, eBioscience, USA) or mouse monoclonal anti-Pan-Cytokeratin AE1/AE3 antibody (1:100 dilution, abcam, UK), respectively. FITC-labeled goat anti-mice IgG was used to detect bound antibodies. 40-6-Diamidino-2-phenylindole (DAPI) was used for nuclear counterstaining. PHKs were incubated in RPMI 1640 containing 10% FBS for 24 h before fixing. The cells were observed by confocal microscopy (LSM780, Zeiss, Jena, Germany). A negative (no antibody) control was included.

### Small interfering RNA (siRNA) transfection

The C/EBPβ-targeting oligonucleotide was designed based on the full-length human C/EBPβ cDNA sequence (NM_005194) (Shanghai Genechem Co. Ltd., Shanghai, China). Three sequences (S1 Table) targeting different regions of the C/EBPβ gene were designed. The first sequence, which matches the sequence located at nucleotides 1448–1466 of the C/EBPβ cDNA, was the most effective and was used to knock down endogenous C/EBPβ in the following experiments. A nonsilencing-siRNA (S1 Table) was used as a negative control.

The transfection process was performed according to the Lipofectamine 2000 instructions. Briefly, 24 h before transfection, cells were grown in six-well plates and the media was changed with 800 μL growth media supplemented with serum, but without antibiotics. In addition, 6 μL of 20 μM siRNA or 4 μL of Lipofectamine 2000 was added to 100 μL of 1640 media without serum and incubated for 5 min at room temperature separately before mixing. The mixture was incubated for 20 min at room temperature before it was added to the cells, and the final concentration of siRNA was 120nM. Eight hours later, media was replaced with fresh complete media without antibiotics. Cells were collected after 48 h for further study.

### Lentiviral construction and infection

For the TNIP1 shRNAs, four self-complementary oligonucleotides carrying shRNAs against human *TNIP1* (NM_006058.4) were designed (Shanghai Sunbio, Shanghai, China) (S2 Table). shRNA #4, which had a targeted gene sequence located in the homologous region of *TNIP1*, was effective at decreasing the *TNIP1* mRNA expression level and was used in the remaining experiments. The green fluorescent protein (GFP) tagged pMagic 4.1 lentiviral vectors and the red fluorescent protein (RFP) tagged pMagic 5.1 lentiviral vectors (Shanghai Sunbio). The GFP-tagged lentivirus was used in cell studies and the percentage of GFP-positive cells reflected the infection efficiency. The RFP-tagged lentivirus was used in animal experiments, and the red fluorescence observed in mice skin reflected the success of TNIP1 shRNA infection *in vivo*. Both vectors were driven by the RNA polymerase III specific promoter hU6. The AgeI and EcoRI sites in pMagic 4.1 and pMagic 5.1were used to introduce annealed shRNA oligonucleotides. To insert the shRNA oligonucleotides, 600 pmoles of each of the two oligonucleotides were annealed in 50 μL of annealing buffer (0.1 M NaCl, 10 mM Tris HCl, pH 7.6) by heating the solution to 95°C and then cooling to room temperature. 10 nM of annealed shRNA oligonucleotides was ligated to 120 ng of vectors in a 20 μL reaction containing 1 μL of T4 DNA ligase (Fermentas) overnight at 16°C. These vectors were verified by sequencing and by PCR (Primer sequences see S3 Table).

For recombinant TNIP1 (rTNIP1), the human *TNIP1* (NM_006058.4) sequence was amplified by PCR from a cDNA template, which was generated from the mRNA of 293 cells grown under standard conditions using the primers of TNIP1-EcoR I (S3 Table). This product was cloned into the pLVX-EGFP-3FLAG lentiviral vector with EcoR I as the only restriction enzyme site upstream of the extrinsic GFP gene. The negative control oligonucleotides are shown in S2 Table (Shanghai Sunbio).

Lentiviruses were generated by co-transfecting 20 μg of recombinant lentiviral vector, 15 μg of pHelper vector 1.0, and 10 μg of pHelper vector 2.0 into 293T cells using a transfection reagent (Shanghai Sunbio). Supernatants containing lentiviral particles were collected 48 h after transfection, filtered through a 0.45μm membrane, and concentrated by ultracentrifugation (4°C, 82700×g, 2h).

HaCaT cells were infected by lentiviral particles according to the manufacturer’s recommendations. Briefly, 24 h before infection, cells were grown in six-well plates in 2 ml of complete media without antibiotics. 15–30 μL of viral stock (MOI = 20) and 20 μL of 1 mg/mL polybrene were added to the cells and incubated at 37°C in a 5% CO_2_-humidified incubator. Finally media was changed with fresh complete media at 12 hours post-infection. Cells that stably expressed rTNIP1 or TNIP1 shRNA were obtained and validated by cell sorting using flow cytometry. Selected clones were frozen in liquid nitrogen after expansion before further use.

PHKs were infected by the same protocol as described for HaCaT cells with slight modifications. At 12 h post-infection, cells were washed with phosphate buffered saline and maintained in defined keratinocyte-serum free media. After expansion in culture for 72 h, the cells were used for *in vitro* assays.

### Real-time quantitative reverse transcription–polymerase chain reaction (qRT-PCR)

Total RNA was extracted from human epidermis or HaCaT cells using TRIzol reagent (Invitrogen, USA) according to the manufacturer’s protocol. RNAse-free DNase I was used to eliminate DNA contamination. qRT-PCR was performed according to standard methods. The primers used in qRT-PCR are shown in S3 Table. The relative quantification was obtained using the ^ΔΔ^CT method relative to a reference gene (β-actin).

### Western blotting

Total protein was prepared from cells or dispase-separated epidermis and then quantified by the Bradford method. Briefly, the detergent-soluble fractions were subjected to SDS-PAGE according to a standard protocol. Separated protein bands were transferred to a nitrocellulose filter. Membranes were blocked with 5% skim milk powder at room temperature for 2 h and incubated overnight with primary antibody at 4°C. Detection of the secondary antibody was performed using the ECL Plus kit (Amersham Pharmacia, Uppsala, Sweden) according to the manufacturer’s instructions. Primary antibodies used in Western blotting included mice monoclonal anti-TNIP1 (1:1000 dilution, USA), rabbit polyclonal anti-cytokerotin (CK) 6 (1:1000 dilution, GeneTex), rabbit polyclonal anti-C/EBPβ (1:1000 dilution, GeneTex), rabbit polyclonal anti-Erk1/2(1:1000 dilution, GeneTex), rabbit monoclonal anti-p-Erk1/2 (1:1000 dilution, Cell Signaling Technology, USA), and rabbit polyclonal anti-TNIP1 (1:1000 dilution, LSBio, USA) antibodies. HRP-conjugated goat anti-mouse IgG and goat anti-rabbit IgG were obtained from Santa Cruz Biotechnology (Santa Cruz, CA, USA). The images were captured and analyzed using Quantity One software.

### Immunohistochemistry (IHC)

Serial 4-μm thick sections from formaldehyde-fixed paraffin-embedded skin tissue were deparaffinized in 100% xylene and rehydrated in descending dilutions of ethanol and water washes. Heat-induced antigen retrieval was performed, followed by endogenous peroxidase activity and non-specific antigen blocking with 3% hydrogen peroxide and serum, respectively. Primary antibodies used in IHC staining included mice anti-human TNIP1 (1:200 dilution, eBioscience), rabbit polyclonal anti-IκBα (1:100 dilution BOSTER, China), rabbit polyclonal anti-TNFα (1:100 dilution, BOSTER), rabbit anti-mice IL-1a (1:100 dilution, BOSTER), and rabbit polyclonal anti-IL-1b (1:100 dilution, BOSTER). Then samples were subsequently incubated with biotin-labeled rabbit anti-mice or goat anti-rabbit antibody, and streptavidin-conjugated HRP (Maixin Inc, China). Sections were visualized with 3, 3’-diaminobenzidine and counterstained with hematoxylin.

The IHC results were evaluated and scored independently by two pathologists in a semiquantitative manner. The percentage scoring of staining positive cells was as follows: 0 (0%), 1 (1–10%), 2 (11–50%), and 3 (>50%). The staining intensity was visually scored and stratified as follows: 0 (negative), 1 (weak), 2 (moderate), and 3 (strong). A final immunoreactivity score was obtained for each case by multiplying the percentage and the intensity score.

### Co-Immunoprecipitation (Co-IP)

HaCaT cells were collected and lysed in ice-cold Pierce IP lysis buffer (Thermo Scientific, USA). TNIP1 complex was immunoprecipitated with anti-TNIP1 antibody (1:1000 dilution, eBioscience) and detected with anti-Erk2 antibody (1:1000 dilution, GeneTex). The Erk2 complex was immunoprecipitated with anti-Erk2 antibody and detected with anti-TNIP1 antibody. Protein A-Sepharose beads were added to bind the complex from solution. The complex was brought down in the pellet by centrifugation and boiled in the presence of SDS to liberate antigen. The immunoblotting procedures were the same as described above. Naive IgG was used as a negative control.

### Cell Counting Kit-8 (CCK-8) assay

A CCK-8 proliferation assay kit (Dojindo, Kumamoto, Japan) was used to analyze cell viability according to the manufacture’s instruction. Briefly, cells were cultured in 96-well plates (Collagen IV-coated 96-well plates for PHKs, the below is same) and incubated with 10 μL CCK-8 solution in 100 μL of fresh media for 3 h at 37°C. The absorbance at 450 nm was detected after incubation.

### BrdU assay

A Cell Proliferation ELISA BrdU colorimetric kit (Roche Applied Science, Penzberg, Germany) was used to determine the quantity of BrdU incorporated into cells, which is a direct indication of cell proliferation, according to the manufacturer’s protocol. Briefly, cells were cultured in 96-well plates and incubated with BrdU for 2 h at 37°C. Then, the cells were fixed and the DNA was denatured by adding FixDenat solution (Roche). The anti-BrdU peroxidase conjugated antibody was added for 45 min at room temperature, and then the cells were rinsed. Immune complexes were detected by adding substrate solution and stop solution at an absorbance of 450 nm.

### Mice and treatments

BALB/c mice were purchased from the laboratory animal center of the Third Military Medical University (Chongqing, China) and were maintained under specific pathogen-free conditions according to standard laboratory procedures.

Mice, 8 to 10 weeks of age, received intradermal back injections of RFP-tagged lentiviral particles encoding TNIP1 shRNA (7.5×10^7^ TU, 150 μL) or control shRNA (7.5×10^7^ TU, 50 μL) on their back skin. A whole-body optical imaging system (CRi's NEW Maestro EX In-Vivo Imaging System, CRi Inc., Woburn, MA, USA) was used to detect red fluorescence seven days after the intradermal injections. An excitation wavelength of 630 nm and an emission wavelength of 800 nm were used. Skin biopsies around the injection sites were collected to detect TNIP1 expression by Western blotting, and to detect IκBα, TNFα, IL-1a and IL-1b expression by IHC staining.

IMQ treatments were performed as previously described [24, 33]. Mice received a daily topical IMQ cream (5%) (Mingxin, Sichuan, China) or lotion control (Ctrl) for six consecutive days. Skin inflammation was scored using a previously described scoring system [24, 33]. Histological sections were prepared by the Pathology Room of Dermatology Department of Southwest Hospital.

### Statistical analysis

Data were analyzed using SPSS 13.0 software, and results are expressed as mean ± standard deviation (SD) or mean ± standard error of the mean (SEM). Statistical differences between the groups were assessed by one-way analysis of variance (ANOVA) followed by Duncan’s Multiple Range test. *, p<0.05; **, p<0.01.

## Results

### TNIP1 expression was decreased in psoriatic lesions compared with normal skin

**Fig 1:**
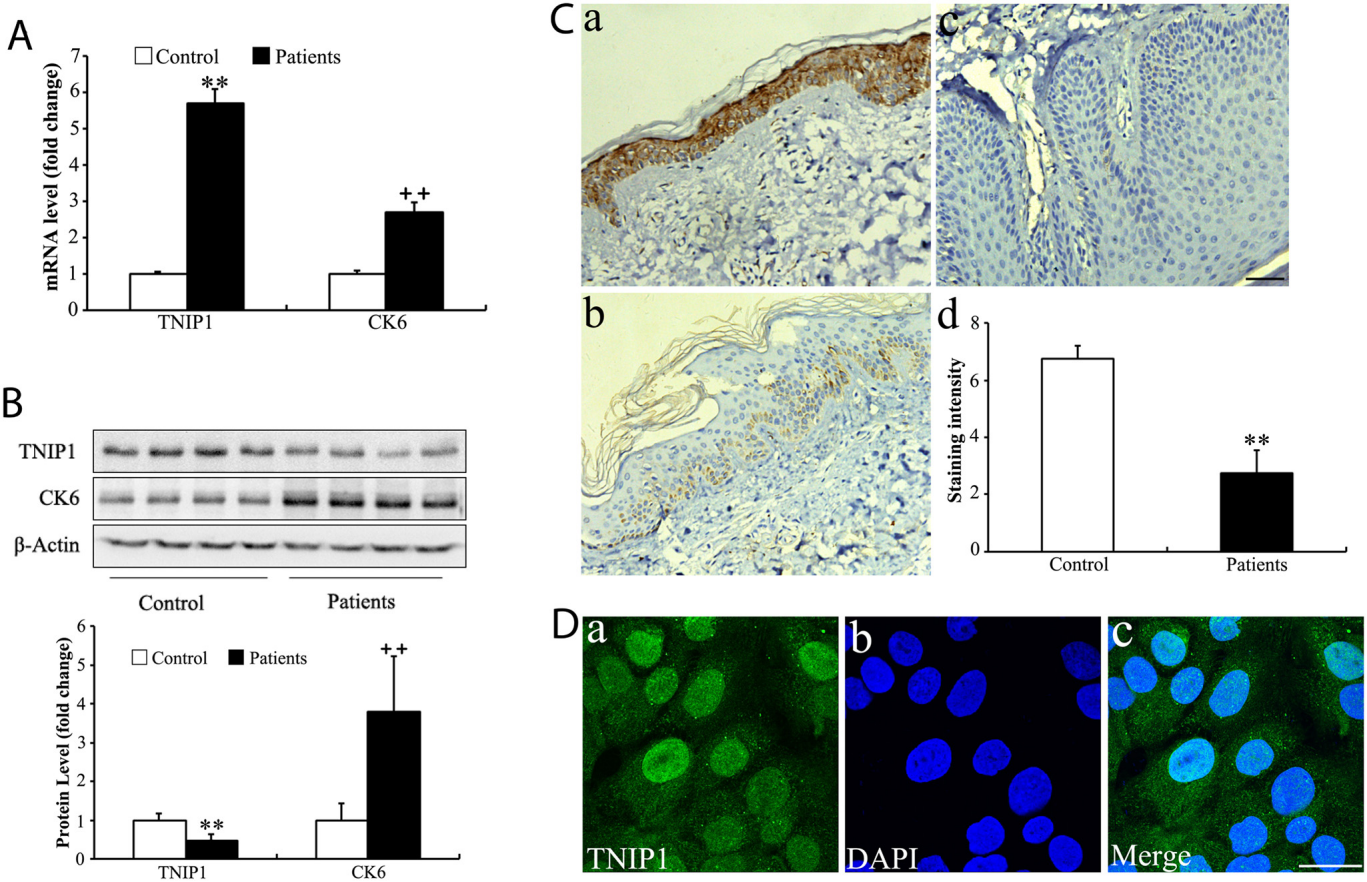
*TNIP1*/TNIP1 and CK6 expression in psoriatic lesions. (A) Expression of *TNIP1* and CK6 mRNA levels based on qRT-PCR in the epidermis of psoriatic plaque of patients with moderate to severe plaque psoriasis (n = 12) and the epidermis of normal controls (n = 12). Data shown represent mean ± SD; (B) Expression of TNIP1 and CK6 protein levels based on Western blotting in psoriatic plaque (n = 4) and controls (n = 4) skin. Data shown represent mean ± SD. (C) Tissue sections obtained from patients with psoriasis (n = 8; panel b and panel c) or from healthy controls (n = 8; panel a) were stained with mice anti-TNIP1 antibody and counterstained with hematoxylin. Bar = 100 μm. Staining intensity was scored in a semiquantitative manner by two independent observers. Data are presented as mean staining intensity grade ± SEM. (D) The distribution of TNIP1 in HaCaT cells identified by immunofluorescence staining. Bar = 30 μm. *, p<0.05; **, p<0.01.

*TNIP1* was previously shown to be significantly different between psoriatic skin and normal skin [6]. To quantify the expression level of *TNIP1* /TNIP1 specifically in the epidermis, we separated the epidermis and dermis using Dispase treatment. qRT-PCR was performed to examine the expression level of *TNIP1* in the epidermis of psoriatic (n = 12) and control (n = 12) biopsies. *TNIP1* expression was increased 5.7-fold in the epidermis of psoriatic biopsies compared with normal skin sections (Fig 1A). Next, we collected four skin biopsy samples from moderate to severe plaque psoriasis patients and from four healthy controls. Using Western blotting, there was a 2-fold reduction of TNIP1 in the epidermis of psoriatic biopsies (Fig 1B). In contrast, the expression of CK6, a marker of epidermal proliferation, was increased at both the mRNA (2.7-fold increase) (Fig 1A) and protein levels (3.8-fold increase) (Fig 1B) in the epidermis of psoriatic biopsies.

In order to identify which cell type(s) in the skin were responsible for the reduced expression of TNIP1, we examined the expression of TNIP1 protein in a series of psoriatic (n = 8) and control (n = 8) biopsies using IHC (Fig 1C). TNIP1 was expressed strongly throughout the normal epidermis (Fig 1C, panel a), whereas its expression was either very weak (Fig 1B, panel b) or completely absent (Fig 1B, panel c) in psoriatic epidermis samples. In addition, TNIP1 was mainly expressed in the cytoplasm and nucleus of keratinocytes in both normal and psoriatic samples. The same cellular distribution was observed in a spontaneously immortalized human keratinocyte line (Fig 1D), which was in line with a previous study by Gurevich et al [12]. Our data indicated that decreased TNIP1 expression in psoriatic skin results from its reduced expression in keratinocytes, potentially leading to abnormal proliferation and differentiation.

### Effects of altering TNIP1 expression on keratinocyte proliferation

**Fig 2:**
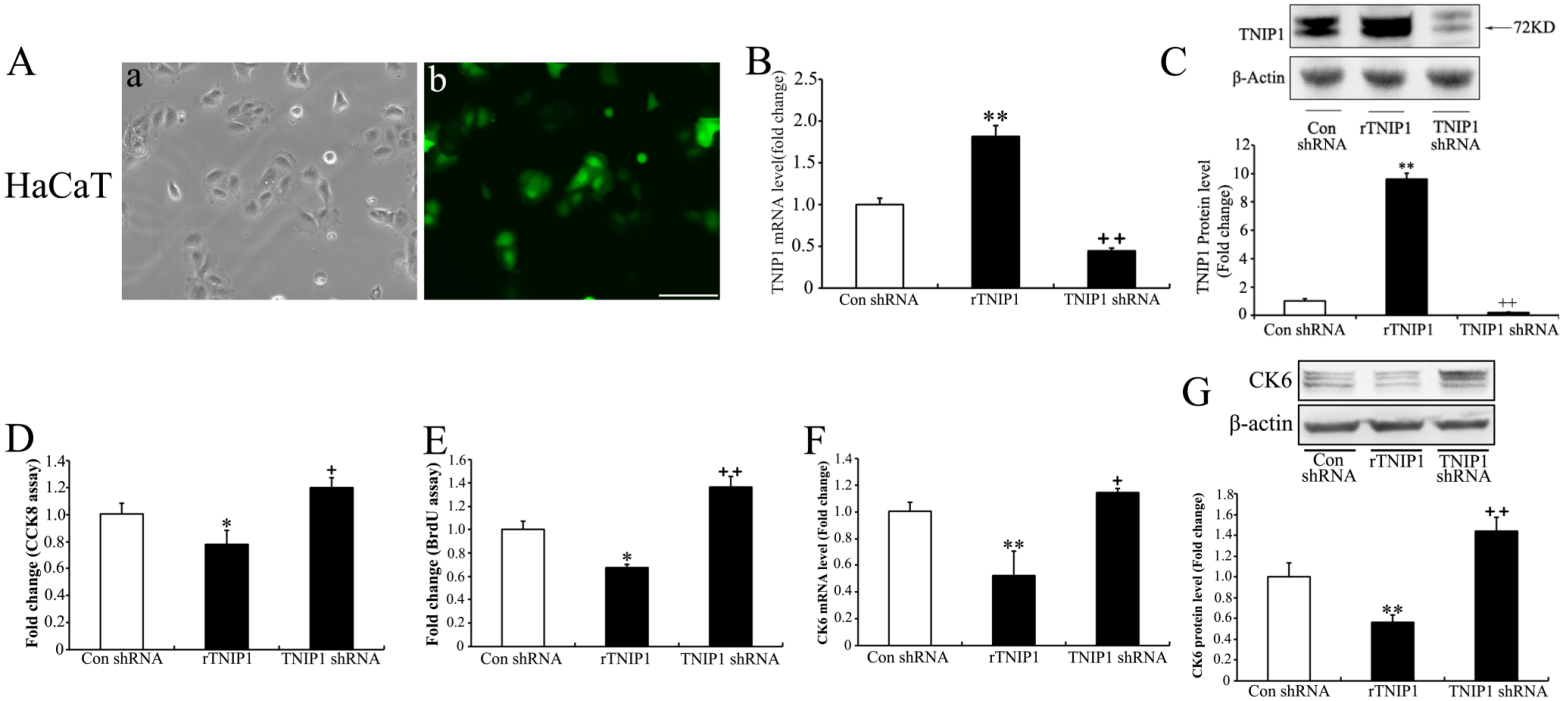
**Upregulation or downregulation of *TNIP1*/TNIP1 expression in HaCaT cells and the anti-proliferative effect of TNIP1 on HaCaT cells** (A) Green fluorescence observed in HaCaT cells stably infected by lentiviral particles respectively encoding rTNIP1, TNIP1 shRNA or a non-specific shRNA (control shRNA) as control. Bar = 100 μm. (B) mRNA expression levels of *TNIP1* in HaCaT cells confirmed by qRT-PCR. (C) The increase of TNIP1 in rTNIP1 infected HaCaT cells and the decrease of TNIP1 in shRNA infected HaCaT cells was confirmed by Western blotting. (D) Cell viability was assessed using the CCK-8 assay in rTNIP1, TNIP1 shRNA or control shRNA HaCaT cells. (E) HaCaT Cells stably infected with rTNIP1, TNIP1 shRNA, or control shRNA were assessed using the BrdU assay. (F, G) CK6 mRNA and protein levels were assessed by qRT-PCR and Western blotting, respectively, in rTNIP1, TNIP1 shRNA or control shRNA infected HaCaT cells. Data shown represent mean ± SD of three independent experiments performed in duplicates. *, p<0.05; **, p<0.01.

We used lentiviral vectors encoding TNIP1 shRNA in the HaCaT cell line, which has been shown to express endogenous TNIP1 (Fig 1D), to downregulate the expression level of TNIP1. To upregulate the expression of TNIP1, the lentiviral vector containing cDNA of the full-length human *TNIP1* gene (NM_006058.4) (rTNIP1) was used. The same unspecific shRNA (control shRNA) was employed as control. Green fluorescence was observed in HaCaT cells 72 h post infection (Fig 2A). Downregulation of TNIP1/ *TNIP1* expression levels in HaCaT cells was both confirmed at the gene level (2.5-fold decrease) (Fig 2B) and protein level (4.8-fold decrease) (Fig 2C) by qRT-PCR and Western blotting, respectively. Infection of rTNIP1 in HaCaT cells led to a 2.3–fold increase in the mRNA level (Fig 2B) and a 9.6-fold increase in the protein level of TNIP1 (Fig 2C).

In order to determine the effects of TNIP1 expression on keratinocyte proliferation, we measured cell viability and proliferation using the CCK-8 assay and BrdU incorporation. TNIP1 downregulation increased cell proliferation (Fig 2D and 2E), and the CK6 expression level was upregulated to 114% of the control gene level (Fig 2F) and 144% of the control protein level (Fig 2G), which was consistent with an increase in cell proliferation. TNIP1 upregulation led to decreased cell viability (Fig 2D) and reduced BrdU incorporation into cells (Fig 2E). Furthermore, the CK6 expression level decreased (Fig 2F and 2G).

**Fig 3:**
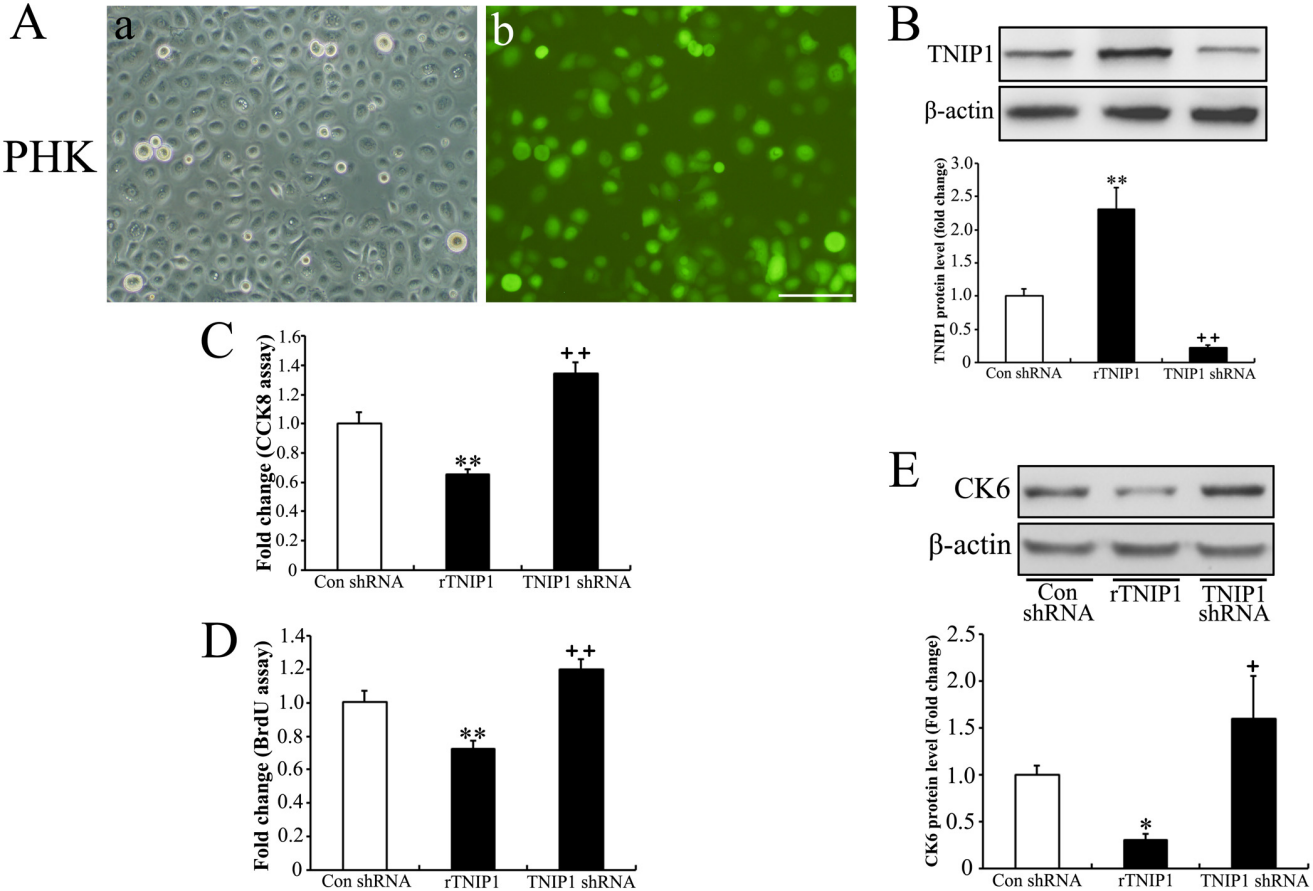
Alteration of TNIP1 expression in PHKs and the anti-proliferative effect of TNIP1 on PHKs. (A) Green fluorescence observed in PHKs stably infected by lentiviral particles respectively encoding rTNIP1, TNIP1 shRNA or a non-specific shRNA (control shRNA). Bar = 100 μm. (B) Alteration of TNIP1 in PHKs is confirmed by Western blotting. PHKs stably infected with rTNIP1, TNIP1 shRNA, or control shRNA were assessed using (C) the CCK-8 assay, (D) the BrdU assay and (E) Western blotting (for CK6 protein level). Data are presented as mean ± SD of three independent experiments (*P<0.05 or **P<0.01 compared with control cells).

We then confirmed these results in PHKs (Fig 3A and 3B) using the CCK8 (Fig 3C) and the BrdU incorporation assays (Fig 3D). The CK6 expression was found to be concomitant with the proliferation rate (Fig 3E).

### The role of TNIP1 on the Erk1/2 and C/EBPβ signaling pathways

**Fig 4:**
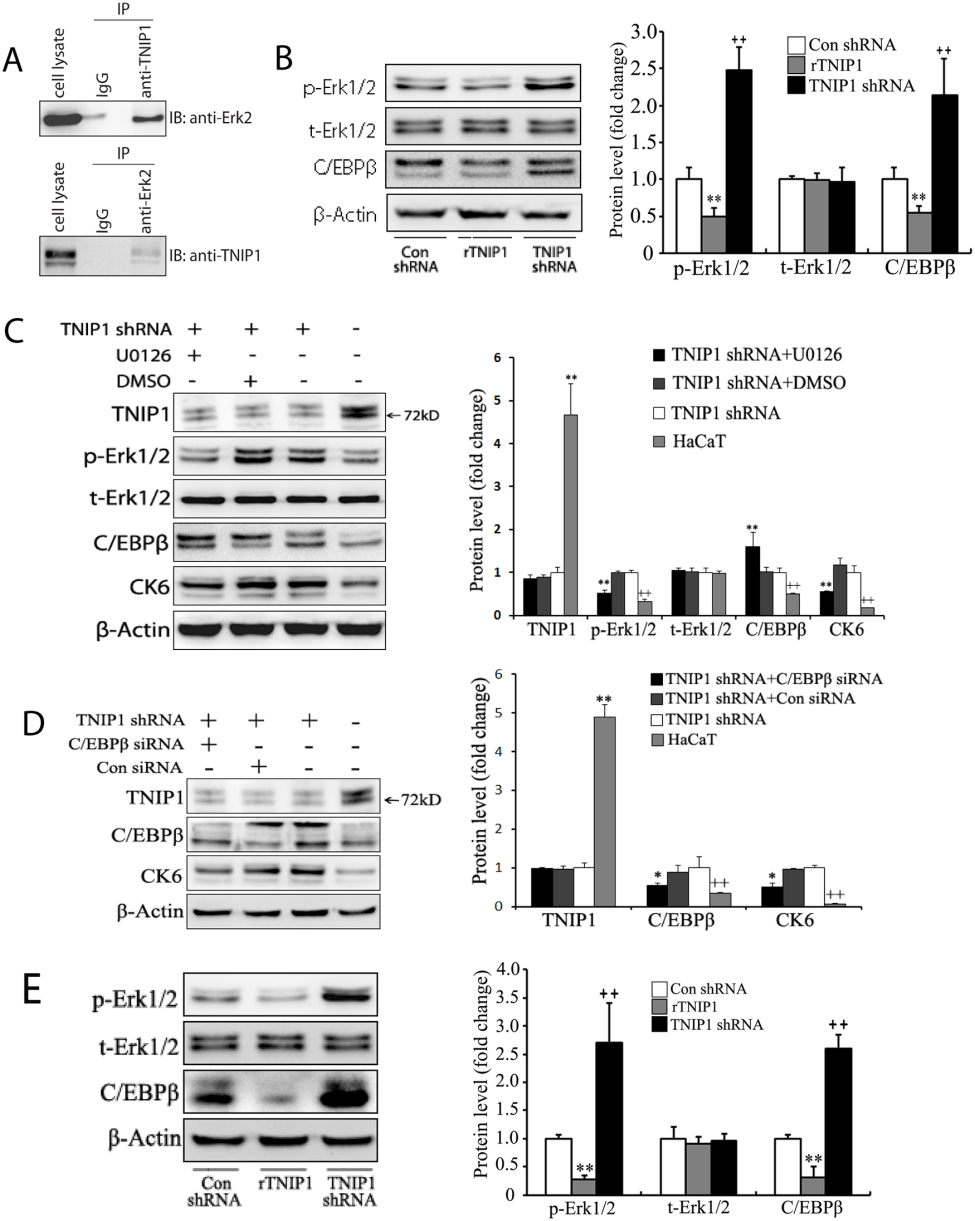
TNIP1 modulates the proliferation of keratinocytes by targeting Erk1/2 and C/EBPβ. (A) The interaction of TNIP1 and Erk2 in HaCaT cells was detected by Co-IP; (B) Downregulated TNIP1 resulted in the upregulation of p-Erk1/2 and C/EBPβ in HaCaT cells. (C) The pro-proliferative effect of downregulated TNIP1 in HaCaT cells was abrogated by U0126, an inhibitor of Erk1/2. TNIP1 shRNA HaCaT cells were cultured in the absence or presence of 20 μM U0126 (Beyotime, Shanghai, China) for 12 h. Dimethyl sulfoxide (DMSO) was used as control. Western blotting was performed to detect TNIP1, total Erk-1/2 (t-Erk1/2), phospho-Erk-1/2 (p-Erk1/2), C/EBPβ and CK6. (D) The pro-proliferative effect of downregulated TNIP1 in HaCaT cells was abrogated by C/EBPβ targeting siRNA. TNIP1 shRNA HaCaT cells were incubated in the absence or presence of 120 nM C/EBPβ targeting siRNA (Shanghai Genechem Co. Ltd.) for 8 h. A scrambled siRNA was used as control siRNA. Western blotting was performed to detect TNIP1, C/EBPβ and CK6 at 48 post-transfection. (E) Downregulated TNIP1 resulted in the upregulation of p-Erk1/2 and C/EBPβ in PHKs. Data are presented as mean ± SD of three independent experiments (*P<0.05 or **P<0.01 compared with control cells).

Previous studies have shown that TNIP1 interacts with Erk2 and attenuates Erk2 signals in TNIP1-expressing Saos-2 cells [34]. We performed Co-IP and confirmed that TNIP1 interacts with Erk2 in HaCaT cells (Fig 4A). Western blotting showed that the phosphorylation level of Erk1/2 (p-Erk1/2) was upregulated to 248% of control in TNIP1 shRNA HaCaT cells and downregulated to 50% of control in rTNIP1 HaCaT cells, while total Erk1/2 (t-Erk1/2) remained the same level (Fig 4B). TNIP1 downregulation promoted Erk1/2 phosphorylation, indicating enhanced Erk1/2 activation. Conversely, overexpression of TNIP1 suppressed Erk1/2 phosphorylation.

To investigate the molecular pathways associated with CK6 upregulation in HaCaT cells, U0126 (Beyotime, Shanghai, China) was used to selectively inhibit Erk1/2 signaling. TNIP1 shRNA infected HaCaT cells were pre-incubated with U0126 (20 μM) for 12 h. CK6 expression level was assessed with Western blotting (Fig 4C). U0126 significantly reduced the expression of CK6 to 55% of control (TNIP1 shRNA HaCaT cells), but was still higher than that in HaCaT cells, confirming that the Erk1/2 pathway was involved in TNIP1 regulation of CK6 expression. DMSO showed no significant effect on Erk1/2 or CK6. Interestingly, we found that inhibition of p-Erk1/2 in TNIP1 shRNA HaCaT cells increased the level of C/EBPβ to 160% of that in TNIP1 shRNA HaCaT cells (Fig 4C), which was consistent with a previous study that showed C/EBPβ could be negatively regulate by phosphorylation of Erk in the IL-17R signaling pathway [35]. However, there may be other factors contributing to the regulation of CK6 by Erk1/2 in TNIP1 knock-down HaCaT cells.

TNIP1 has been shown to selectively control C/EBPβ activity in primary immune cells [21]. We detected the expression level of C/EBPβ in TNIP1-altered HaCaT cells by Western blotting. TNIP1 downregulation led to 2.14-fold increase in C/EBPβ expression (Fig 4B), indicating that the C/EBPβ pathway was activated in TNIP1 shRNA infected HaCaT cells. Conversely, overexpression of TNIP1 decreased C/EBPβ expression to 55% of control (Fig 4B).

Furthermore, we knocked down the expression of endogenous C/EBPβ via C/EBPβ-targeting siRNA in TNIP1shRNA infected HaCaT cells. A scrambled siRNA was used as the control. The cells were collected 48 hours post-transfection, and the CK6 expression level was detected by Western blotting. The expression level of C/EBPβ protein was decreased by 40% following siRNA knockdown when compared with that treated with control (p = 0.04), but was higher than that of HaCaT cells. However, this difference rather represented a trend, since it was not significant (p = 0.18). Notably, the control siRNA demonstrated an unspecific effect as it suppressed the C/EBPβ level to 88% of the untreated TNIP1 shRNA HaCaT cells (p = 0.41). C/EBPβ knockdown prominently reduced CK6 expression levels to 51% of control (TNIP1 shRNA HaCaT cells), but was still higher than that in HaCaT cells, which was 7% of control (Fig 4D), indicating that C/EBPβ was an important intermediator between TNIP1 and CK6. Similar results were also obtained in PHKs (Fig 4E).

### Decreased TNIP1 expression exaggerated psoriatic conditions in an IMQ-induced mice model

**Fig 5:**
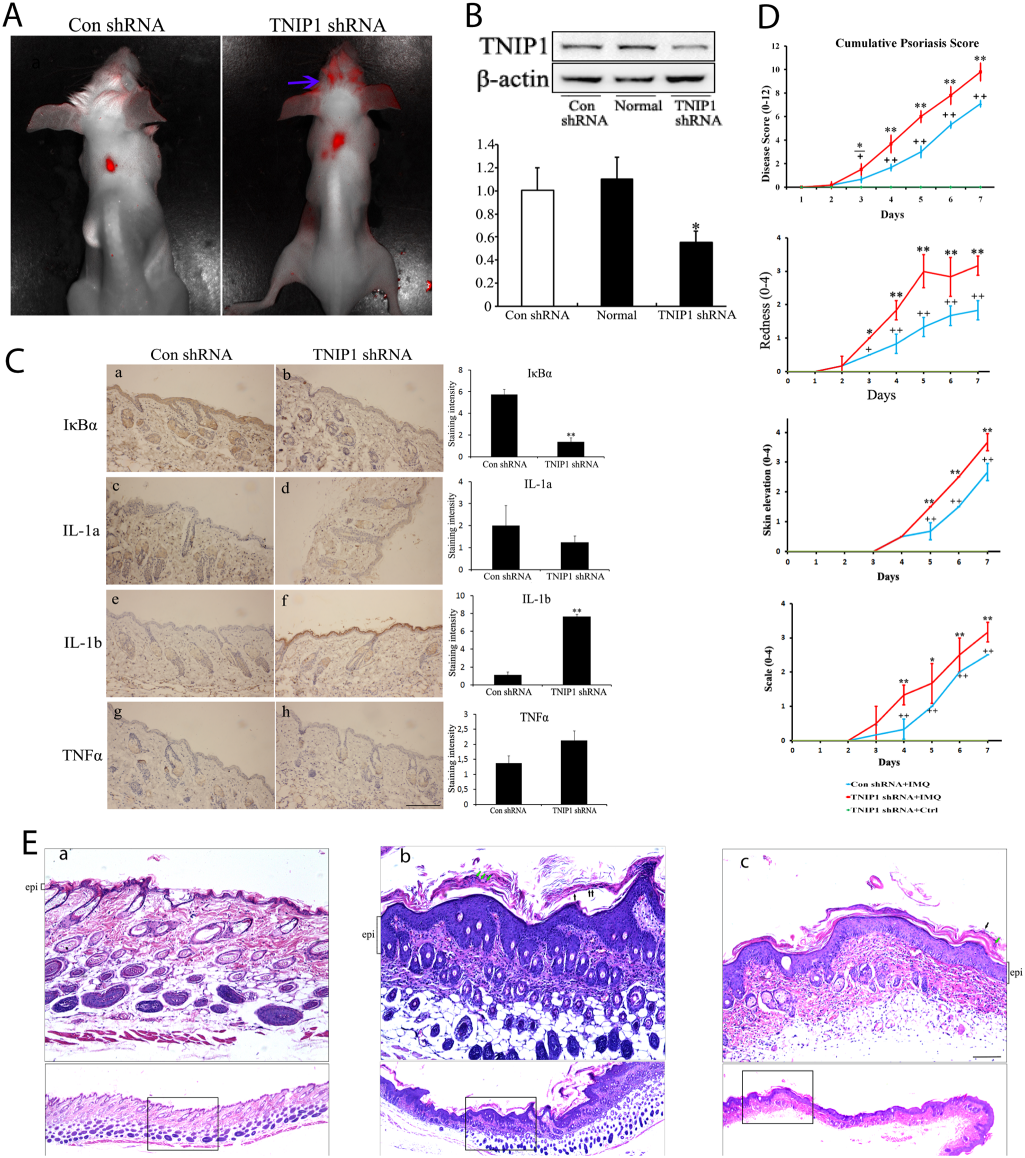
Downregulation of TNIP1 exaggerates IMQ-induced psoriasis-like dermatitis *in vivo*. (A) Red fluorescence observed in mice seven days after intradermal injections of lentiviral particles encoding TNIP1 shRNA or control shRNA, respectively. Blue arrows indicate the unspecific fluorescence of mice hair. The back of the mice had been shaved. (B) Western blotting was performed to verify that the TNIP1 expression level was decreased around the injection site. (C) Skin sections obtained seven days after injection from control shRNA mice (n = 4) or from TNIP1 shRNA mice (n = 4) were stained with rabbit anti-IκBα antibody, rabbit anti-TNFα antibody, rabbit anti-IL-1a antibody and rabbit anti-IL-1b antibody, respectively. Bar = 200 μm. Staining intensity was scored in a semiquantitative manner by two independent observers. Data are presented as mean staining intensity grade ± SEM. (D) Clinical scores of IMQ-induced psoriasis-like dermatitis in control shRNA or TNIP1 shRNA-injected mice at the indicated days with or without IMQ treatment. (E) H&E-stained sections of back skin from the mice at the indicated areas treated with or without IMQ. The epithelial layer (epi) is indicated by brackets. Green arrows indicate neutrophil infiltration and black arrows indicate parakeratosis. The epidermal layer thickness was more prominent, but unevenly distributed in TNIP1 shRNA-injected mice (panel b) compared with control shRNA-injected mice (panel c). No obvious epidermal hyperplasia was observed in TNIP1 shRNA-injected mice treated with topical emollient (the control, panel a). Bar = 100 μm. Data are presented as mean ± SD of three independent experiments (* P<0.05 or **P<0.01 compared with controls).

To investigate the effect of TNIP1 downregulation *in vivo*, we intradermally injected RFP-tagged TNIP1 shRNA lentiviral particles in mice skin. Previous studies have shown that lentiviral-mediated silencing can lead to stable and long lasting RNAi-based gene knockdown [36, 37]. In addition, intradermal injection is efficient for lentiviral gene delivery to the skin [38]. Although the sequence of shRNA against *TNIP1* was designed based on the human gene sequence (NM_006058.4) in our study, mice were successfully infected, as confirmed by whole-body optical imaging (Fig 5A). Moreover, the efficiency of this shRNA against TNIP1 was confirmed by Western blotting using tissues around the injection area seven days post injection (Fig 5B). Knock-down by TNIP1 shRNA led to increased NF-κB activity, as demonstrated by downregulation of IκBα (Fig 5C). It also significantly increased IL-1b expression levels, but had no obvious effect on TNFα or IL-1a expression levels, as demonstrated by IHC staining (Fig 5C).

To investigate whether downregulation of TNIP1 expression could increase the severity of psoriasis, we used a widely accepted IMQ-induced psoriasis-like mice model, which is clinically and pathologically similar of human psoriasis [33]. Briefly, 8–10 week old BALB/c mice received an intradermal injection of lentiviral vectors encoding TNIP1 shRNA to downregulate TNIP1 expression levels. After seven days, IMQ was topically applied daily to induce psoriatic lesions. Treatment of mice with IMQ led to markedly increased redness, scaling, and skin elevation in TNIP1 shRNA infected mice compared with control mice (Fig 5D). This was probably due to the proliferation of keratinocytes and leukocyte infiltration into the skin. In fact, histological examination of skin sections from TNIP1 shRNA treated mice showed significant epidermal hyperplasia (Fig 5E, panel b). Hypogranulosis, hyperkeratosis, and parakeratosis with neutrophils were also observed more severely in shRNA treated mice (Fig 5E, panel b). No significant clinical responses (Fig 5D) or pathological changes (Fig 5E, panel a) were observed after the TNIP1 shRNA treated mice were treated with topical emollient (the control). Hence, downregulated TNIP1 expression in the skin increases the susceptibility to experimental psoriasis in mice.

## Discussion

In this study, we demonstrated that TNIP1 was significantly downregulated in psoriatic epidermis and that TNIP1 modulates keratinocyte proliferation. Besides its pivotal role in anti-inflammation, TNIP1 also regulates keratinocyte growth.

TNIP1 is a widely expressed ubiquitin protein that plays important roles in many physiological and pathological processes in humans. In particular, TNIP1 has been implicated in inflammation and autoimmunity, and its endogenous targets include A20 [14], Erk2 [34], retinoic acid receptor-α and—γ [22], peroxisome proliferator-activated receptors [39], and C/EBPβ [21]. These distinctly different targets suggest broad biological functions of TNIP1. Moreover, TNIP1 expression is increased in esophageal cancer and decreased in prostate cancer [12], suggesting TNIP1 plays different or even opposite roles in different tissue/cell types. We also showed that TNIP1 was distributed in both the cytoplasm and nucleus in epidermal cells and HaCaT cells. This finding is quite different from most other cell types, in which TNIP1 is only expressed in the cytoplasm [12]. These results strongly indicate that further studies are needed to determine the effects of TNIP1 on keratinocytes.

In this study, our results, as well as Nair et al. study [6], showed that the mRNA expression of *TNIP1* was upregulated in psoriatic epidermis. Interestingly, TNIP1 protein was downregulated in psoriatic epidermis, as confirmed by IHC and Western blotting in our study. It is a common phenomenon that mRNA levels do not correlate with protein levels [40][41]. One possible reason for the discrepancy between mRNA and protein levels may be the presence of microRNAs (miRNAs), a class of small non-coding RNAs that bind to target mRNAs and promote transcript degradation and/or inhibit translation [42]. Studies have shown that TNIP1 is a direct functional target of miR-517a/c [43], and the overexpression of miR-486 repressed the expression level of TNIP1 *in vitro* [44]. Interestingly, miR-486 is decreased in psoriatic skin [45], which strongly suggested that there may be other miRNAs contributing to the inhibition of *TNIP1* translation. On the other hand, the increased *TNIP1* mRNA level may be the result of negative feedback regulation of decreased TNIP1 protein levels. However, it is currently unknown whether reduced TNIP1 epidermal expression in psoriasis is caused by transcriptional or post-transcriptional regulation. In this study, we focused on the effect of altered TNIP1 protein levels on keratinocytes.

We found that low TNIP1 expression triggered cell proliferation in both HaCaT cells and PHKs, indicating low TNIP1 expression in psoriatic epidermis may be involved in the pathogenesis of the disease. Indeed, psoriasis is characterized by excessive proliferation of keratinocytes. In addition, psoriatic keratinocytes show a number of proliferation markers, such as CK6 [46], which has been found to be induced by TNIP1 downregulation in our cultured keratinocytes.

Furthermore, we investigated the mechanism of TNIP1-induced keratinocyte proliferation. TNIP1 has been reported to interact with Erk2 [34]. In our study, we showed that TNIP1 downregulation increased the level of p-Erk1/2 in cultured keratinocytes, which is consistent with Johansen C’s findings that the level of p-Erk1/2 increased in psoriatic skin when compared with normal skin [47]. Erk1/2 are members of the mitogen activated protein kinase super family and play a role in mediating cell proliferation [48]. We also found that the expression of CK6 was partially suppressed by the Erk1/2-specific inhibitor U0126.

TNIP1 also selectively controlled the C/EBPβ signaling pathway [21]. In human keratinocytes, C/EBPβ binds with the CK6 promoter and directly induces the synthesis of CK6 [49]. In this study, we speculated that TNIP1 might affect keratinocyte proliferation by targeting C/EBPβ and sequentially modulating CK6 synthesis. C/EBPβ consists of three different isoforms, including the 38 kDa Full and the 34 kDa liver-enriched transcriptional activator protein LAP isoforms, and the 20 kDa liver-enriched transcriptional inhibitory protein LIP [50]. The ratio of these isoforms varies significantly depending on the cell type and developmental stage and can be altered by different stimuli [51]. In this study we focused on LAP because it is thought to be the most transcriptionally active isoform of C/EBPβ, and TNIP1 selectively controls the levels of LAP [21]. C/EBPβ-targeted siRNA was employed to transiently decrease C/EBPβ, resulting in decreased expression of CK6. Taken together, we concluded that TNIP1 regulated keratinocyte proliferation partially by targeting the Erk1/2 and C/EBPβ signaling pathways.

The IMQ-induced psoriasis-like dermatitis mice model has been widely accepted to study erythema, scales and psoriatic plaque formation. IMQ is a ligand for TLR7 and TLR8, which are mainly expressed by monocytes, macrophages, and dendritic cells [52], and is thought to function via the IL-23/IL-17 axis [33]. In our study, TNIP1 shRNA was used to infect mice skin *in vivo*, which was based on previous studies [38, 53, 54]. We employed a midium dose among those recommended, as a high dose would increase the off-target effect, while a low dose would affect the knock-down efficiency. We found 1.5x10^7^ TU of lentivirus led to no obvious fluorescent signal (data not shown). However, 7.5x10^7^ TU of TNIP1 shRNA led to TNIP1 downregulation, as confirmed by fluorescence and Western blotting. In addition, knockdown of TNIP1 led to increased NF-κB activity and increased levels of IL-1b in mice skin, but did not affect the levels of TNFα or IL-1a (IHC staining). These findings are consistent with the study by Zhou J, et al [21]. The high transgene dose used in this study could cause off-target effects, which mainly depended on the dose of siRNA transfected [55, 56]. In our pre-experiment, as S2 Fig shows, we used siRNA #2 as the template for the construction of TNIP1 shRNA, and 100nM as the siRNA transfection concentration. As an additional control, TNIP1 levels were not affected by equivalent amounts (100nM) of control siRNA treatment (lane 6), thereby excluding an undesired effect.

Our study revealed that mice with decreased TNIP1 were more vulnerable to IMQ-induced psoriasis-like dermatitis. These findings are consistent with Callahan’s study [57]. Most notably, IMQ mice treated with TNIP1 shRNA had a thicker epidermal layer compared with control mice (Fig 5E), indicating increased proliferation of epidermal cells. Interestingly, the skin sections from IMQ mice treated with TNIP1 shRNA had an obvious uneven epidermal layer. This might have been associated with an uneven distribution of lentiviral particles, leading to uneven levels of TNIP1 in mice skin. In addition, we noted that TNIP1 downregulation not only promoted epidermal cell proliferation, but also led to more severe inflammation, as demonstrated by significant neutrophil infiltration, and disturbed keratinocyte differentiation, as demonstrated by retention of nuclei in the stratum corneum (parakeratosis) in mice skin sections (Fig 5E). These findings suggest that TNIP1 may be involved in other mechanisms other than regulating keratinocyte proliferation. The increased production of proinflammatory cytokines and chemokines by hyperproliferative epidermal cells may also contribute to this complicated situation [27]. Our animal study had one limitation that should be noted. TNIP1 is widely expressed throughout the body, and thus other cell types at the injection site could have sensed TNIP1 downregulation. Thus, other cell types could have contributed to the scaling hyperplasia. In fact, Callahan’s study showed that TNIP1 downregulation in dendritic cells led to epidermal hyperplasia [50]. Nevertheless, we confirmed that TNIP1 downregulation in mice skin led to exaggerated psoriasis-like dermatitis. However, to clarify the mechanism of TNIP1 in the pathogenesis of psoriasis, it is necessary to extensively explore the functions of TNIP1 *in vivo*.

Taken together, our results show that TNIP1 inhibits keratinocyte proliferation, which is partially mediated by the inhibition of Erk1/2 and C/EBPβ. Moreover, downregulation of TNIP1 in the skin leads to keratinocyte hyperproliferation in mice. Thus, TNIP1 may be a potential therapeutic target for psoriasis.

## Supporting Information

S1 Fig Pan-Cytokeratin AE1/AE3 expression in PHKs. The distribution of Pan-Cytokeratin AE1/AE3 in PHKs was identified by immunofluorescence staining. Bar = 100μm.
(TIF)

S2 Fig The TNIP1 siRNA selected as the template for the construction of TNIP1 shRNA. TNIP1 protein level was most effectively downregulated by 100nM of siRNA2 (TNIP1 siRNA2-H, lane 8). Data analysis was performed in the group transfected by 100nM siRNA. The lower concentration (L) is 70nM.
(TIF)

S1 File ARRIVE Checklist. (PDF)

S1 Table Oligonucleotides used for the creation of anti-C/EBPβ siRNA. (DOC)

S2 Table Oligonucleotides used for the creation of anti-TNIP1 shRNA expression cassettes. (DOC)

S3 Table Primer sequences used in PCR. (DOC)
