# Supplementary figures and images for: Downregulation of TNIP1 Expression Leads to Increased Proliferation of Human Keratinocytes and Severer Psoriasis-Like Conditions in an Imiquimod-Induced Mouse Model of Dermatitis
Source: PLoS One. 2015 Jun 5;10(6):e0127957. doi: 10.1371/journal.pone.0127957 (PMC4457880; doi:10.1371/journal.pone.0127957)

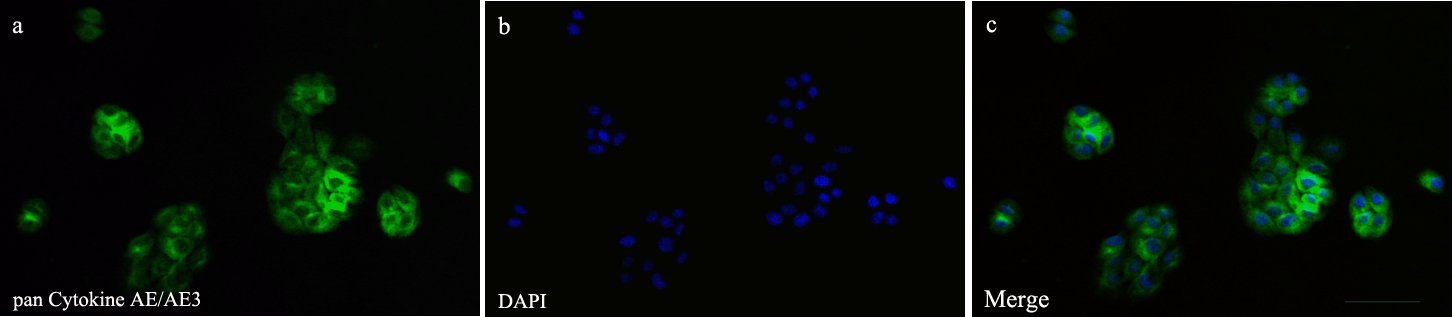

Supplement: S1 Fig — The distribution of Pan-Cytokeratin AE1/AE3 in PHKs was identified by immunofluorescence staining. Bar = 100μm. (TIF) [file pone.0127957.s001.tif]

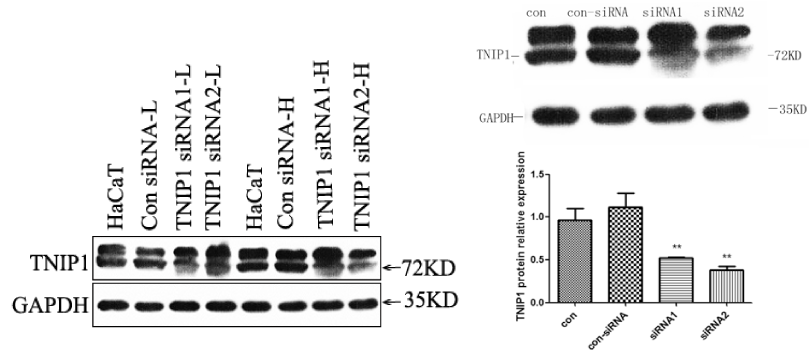

Supplement: S2 Fig — TNIP1 protein level was most effectively downregulated by 100nM of siRNA2 (TNIP1 siRNA2-H, lane 8). Data analysis was performed in the group transfected by 100nM siRNA. The lower concentration (L) is 70nM. (TIF) [file pone.0127957.s002.tif]
