# Supplementary material for: Downregulation of TNIP1 Expression Leads to Increased Proliferation of Human Keratinocytes and Severer Psoriasis-Like Conditions in an Imiquimod-Induced Mouse Model of Dermatitis
Source: PLoS One. 2015 Jun 5;10(6):e0127957. doi: 10.1371/journal.pone.0127957 (PMC4457880; doi:10.1371/journal.pone.0127957)
Supplement: S1 File — (PDF) [file pone.0127957.s003.pdf]

National Centre for the Replacement Refinement & Reduction of Animals in Research

ARRIVE

The ARRIVE guidelines - Examples
Animal Research: Reporting In Vivo Experiments

Carol Kilkenny[1], William J Browne[2], Innes C Cuthill[3], Michael Emerson[4] and Douglas G Altman[5]

[1]The National Centre for the Replacement, Refinement and Reduction of Animals in Research, London, UK , [2]School of Veterinary Science, University of Bristol, Bristol, UK, [3]School of Biological Sciences, University of Bristol, Bristol, UK, [4]National Heart and Lung Institute, Imperial College London, UK, [5]Centre for Statistics in Medicine, University of Oxford, Oxford, UK.

The ARRIVE guidelines are designed to improve the reporting of animal research. This document demonstrates how the ARRIVE guidelines can be used in practice to report animal research, by providing specific examples for each point of the guidelines. The examples given are from a wide range of research using a range of animal species. Each example has been chosen for meeting the criteria of the checkpoint mentioned and does not imply the entire manuscript complies with the ARRIVE guidelines.

	ITEM	RECOMMENDATION	EXAMPLE
Title	1	Provide as accurate and concise a description of the content of the article as possible.	TNFa–induced protein 3-interacting protein 1 exaggerates imiquimod-induced psoriasis-like dermatitis in mice when downregulated
Abstract	2	Provide an accurate summary of the background, research objectives, including details of the species or strain of animal used, key methods, principal findings and conclusions of the study.	BACKGROUND AND PURPOSE: Psoriasis is a chronic, inflammatory skin disease involving both environmental and genetic factors. GWAS revealed that TNIP1 gene is strongly linked to the susceptibility of psoriasis. We revealed that TNIP1 expression is downregulated in psoriasis skin when compared with normals. Furthermore, we discovered that downregulated TNIP1 increased keratinocyte proliferation while overexpression of TNIP1 decreased keratinocyte proliferation. In the animal experiment, our aim was to evaluate the downregulated TNIP1 whether protected or exaggerated imiquimod (IMQ) induced psoriatic dermatitis in mice.<br>EXPERIMENTAL APPROACH: Mice at 8 to 10 weeks of age received intradermal injections of RFP-tagged lentiviral particles encoding shRNA to TNIP1 (7.5×107TU) or control shRNA (7.5×107TU) on their back skin. 7 d later, starting the topical application of IMQ, for 6 consecutive days, then the mice sacrificed and skin sample around injection area were collected for HE staining. We recorded the clinical manifestation of mice during the treatment of IMQ and have skin sections for pathological examination.

			KEY RESULTS: We have observed that mice injected TNIP1 shRNA showed much more severe inflammation during the consecutive treatment of IMQ, and histological examination of skin sections from these mice showed much more significant epidermal hyperplasia.<br><br>CONCLUSIONS AND IMPLICATIONS: These results highlighted that TNIP1 exaggerated IMQ-induced psoriasis-like dermatitis in mice when downregulated and suggest TNIP1 may be a potential therapeutic target for psoriasis.
**INTRODUCTION**			
Background	3	a. Include sufficient scientific background (including relevant references to previous work) to understand the motivation and context for the study, and explain the experimental approach and rationale.	Psoriasis is a common chronic inflammatory skin disorder affecting 1-2% of the northern American and European populations [1], and the TNIP1 gene, which encodes protein TNIP1, has been revealed to be susceptibility gene of psoriasis [2, 3]. However the mechanisms of how the susceptibility gene and the encoded protein contribute to the pathogenesis of psoriasis remain largely unclear. Psoriasis is characterized by hyperproliferation and aberrant terminal differentiation of keratinocytes, and the role of TNIP1 in proliferation of keratinocyte has never been explored. We revealed that TNIP1 expression is downregulated in psoriasis skin when compared with normals, and discovered that downregulated TNIP1 increased keratinocyte proliferation while overexpression of TNIP1 decreased keratinocyte proliferation. Furthermore we intended to explore whether TNIP1 involved in the pathogenesis of psoriasis especially by targeting keratinocytes in vivo.
		b. Explain how and why the animal species and model being used can address the scientific objectives and, where appropriate, the study's relevance to human biology.	For this purpose, we selected the IMQ-induced psoriatic dermatitis mice model [4, 5], which has been widely accepted to study erythema, scales and psoriatic plaque formation.
Objectives	4	Clearly describe the primary and any secondary objectives of the study, or specific hypotheses being tested.	Therefore, the objective of this study was to determine whether TNIP1 involved in pathogenesis of psoriasis and whether the downregulated TNIP1 protected or exaggerated IMQ induced psoriatic dermatitis in mice.

METHODS			
Ethical statement	5	Indicate the nature of the ethical review permissions, relevant licences (e.g. Animal [Scientific Procedures] Act 1986), and national or institutional guidelines for the care and use of animals, that cover the research.	All animal studies were approved by the animal ethics committee of the Third Military Medical University according to Dutch legislation on animal experiments. Mice were anaesthetized with sodium pentobarbital when necessary, and all efforts were made to minimize suffering.
Study design	6	For each experiment, give brief details of the study design including:<br><br>a. The number of experimental and control groups.<br><br>b. Any steps taken to minimise the effects of subjective bias when allocating animals to treatment (e.g. randomisation procedure) and when assessing results (e.g. if done, describe who was blinded and when).<br><br>c. The experimental unit (e.g. a single animal, group or cage of animals).<br><br>d. A time-line diagram or flow chart can be useful to illustrate how complex study designs were carried out.	There're three groups, 3 in each group.<br><br>Animals were randomized into treatment groups by picking numbers out of a hat.<br><br>In the study, n refers to number of animals.<br><br>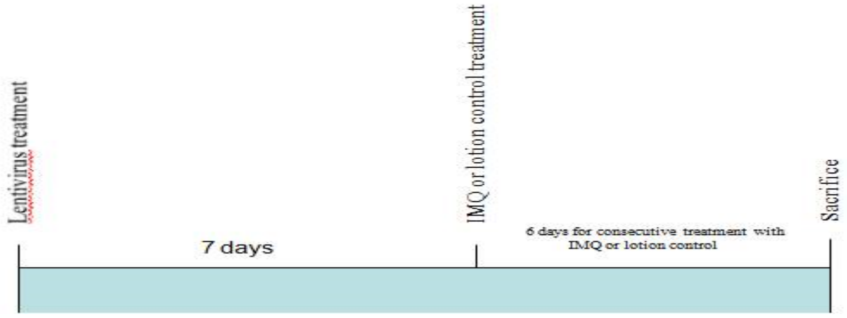

Experimental procedures	7	For each experiment and each experimental group, including controls, provide precise details of all procedures carried out. For example:	
		a. How (e.g. drug formulation and dose, site and route of administration, anaesthesia and analgesia used [including monitoring], surgical procedure, method of euthanasia). Provide details of any specialist equipment used, including supplier(s).	The lentiviral particles (7.5×107TU) were intradermally injected into mice skin via microliter inyeetor(50ul). IMQ cream (5%) was commercial products (Mingxin, Sichuan, China) and was topically applied on back skin of mice, once a day for 6 consecutive days. Mice were anaesthetized with sodium pentobarbital (50mg/kg, i.p.) when necessary.
		b. When (e.g. time of day).	Treatment were conducted at about 10:00 A.M. in the morning.
		c. Where (e.g. home cage, laboratory, water maze).	Experiment were conducted under Laminar Flow Hood in the SPF animal house where the mice were maintained.
		d. Why (e.g. rationale for choice of specific anaesthetic, route of administration, drug dose used).	To observe the clinical manifestation of mice at scheduled timepoint.
Experimental animals	8	a. Provide details of the animals used, including species, strain, sex, developmental stage (e.g. mean or median age plus age range) and weight (e.g. mean or median weight plus weight range).	Male C57BL/6J mice (20g ± 1.4 g), aged 8–10 weeks, were included (n = 9).
		b. Provide further relevant information such as the source of animals, international strain nomenclature, genetic modification status (e.g. knock-out or transgenic), genotype, health/immune status, drug or test naïve, previous procedures, etc.	BALB/c mice were purchased from the laboratory animal center of the Third Military Medical University (Chongqing, China) and Vendor health reports indicated that the mice were free of known viral, bacterial and parasitic pathogens.

Housing and husbandry	9	Provide details of:	
		a. Housing (type of facility e.g. specific pathogen free [SPF]; type of cage or housing; bedding material; number of cage companions; tank shape and material etc. for fish).	Mice were housed with an inverse 12 hours day-night cycle with lights on at 8:30pm in a temperature (21± 2°C) and humidity (55±5%) controlled room.
		b. Husbandry conditions (e.g. breeding programme, light/dark cycle, temperature, quality of water etc for fish, type of food, access to food and water, environmental enrichment).	All mice were allowed free access to water and a maintenance diet containing 0.75% calcium in a 12-hour light/dark cycle, with room temperature at 21±2 °C. All cages contained wood shavings, bedding.
		c. Welfare-related assessments and interventions that were carried out prior to, during, or after the experiment.	All cages contained a cardboard tube for environmental enrichment.
Sample size	10	a. Specify the total number of animals used in each experiment, and the number of animals in each experimental group.	Nine male healthy mice were divided into three groups of three each. Animals of group I received TNIP1 shRNA lentivirus injection and lotion control topical application, group II animals received control shRNA lentivirus injection and IMQ topical application, and group III received TNIP1 shRNA lentivirus injection and IMQ topical application for six days.
		b. Explain how the number of animals was arrived at. Provide details of any sample size calculation used.	We decided the sample size by two groups of completely random design of sample mean comparison. We performed the experiments as before mentioned for we only have 9 mice met the age standard. Since the clinical and pathological menifestation showed the same trend and the difference were significant, which were sufficient to draw our conclusion that downregulated TNIP1 exaggerated IMQ-induced psoriatic dermatitis in mice. So we did not repeat the experiment and n=3.
		c. Indicate the number of independent replications of each experiment, if relevant	The experiment was not repeated due to unnecessary.
Allocating animals to experimental groups	11	a. Give full details of how animals were allocated to experimental groups, including randomisation or matching if done.	The animals were allocated to experimental groups randomly.

		b. Describe the order in which the animals in the different experimental groups were treated and assessed.	The order: intradermal injection of lentiviral particles---7 d later, starting the topical application of IMQ, for 6 consecutive days—mice sacrificed for HE staining.
Experimental outcomes	12	Clearly define the primary and secondary experimental outcomes assessed (e.g. cell death, molecular markers, behavioural changes).	One primary outcome measures was analyzed: Clinical scores of IMQ-induced psoriatic dermatitis. In addition, three secondary outcome measures were evaluated: epidermis thickness, neutrophil infiltration and parakeratosis.
Statistical methods	13	a. Provide details of the statistical methods used for each analysis.	Data were analyzed using SPSS 13.0 software, and results are expressed as mean±standard deviation (SD). Statistical differences between the groups were assessed by one-way analysis of variance (ANOVA) followed by Duncan's Multiple Range test. Differences were considered significant when P<0.05 or P<0.01.
		b. Specify the unit of analysis for each dataset (e.g. single animal, group of animals, single neuron).	For each test, the experimental unit was an individual animal.
		c. Describe any methods used to assess whether the data met the assumptions of the statistical approach.	In the first round of animal experiment, we randomly allocated three mice in each group. During the consecutive 6 days IMQ treatment, the clinical manifestation of mice (erythema, scales, and thickness of skin) showed significant differences between the three groups since the third day. We did not repeat the experiment later because it's already sufficient to draw the conclusion that downregulated TNIP1 exaggerated IMQ-induced psoriatic dermatitis in mice, or the mice would sacrifice unnecessarily.
**RESULTS**			
Baseline data	14	For each experimental group, report relevant characteristics and health status of animals (e.g. weight, microbiological status, and drug or test naïve) prior to treatment or testing. (This information can often be tabulated).	BALB/c mice were purchased from the laboratory animal center of the Third Military Medical University (Chongqing, China) and were maintained under specific pathogen-free conditions according to standard laboratory procedures. The mice were free of all viral, bacterial,and parasitic pathogens, except for Helicobacter species.
Numbers analysed	15	a. Report the number of animals in each group included in each analysis. Report absolute numbers (e.g. 10/20, not 50%[2]).	There are three groups, and n=3 in each group.

		b. If any animals or data were not included in the analysis, explain why.	No animals or data were not included.
Outcomes and estimation	16	Report the results for each analysis carried out, with a measure of precision (e.g. standard error or confidence interval).	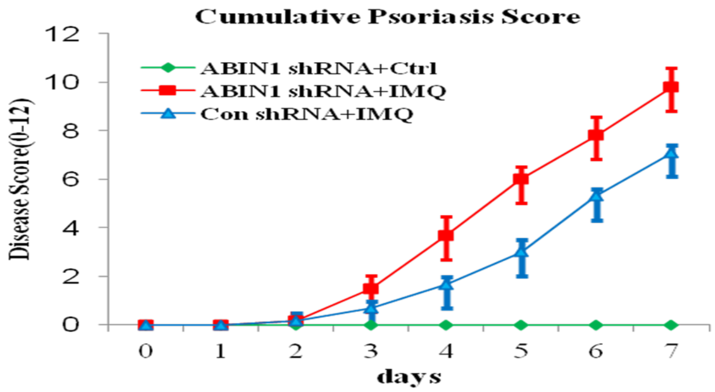
Adverse events	17	a. Give details of all important adverse events in each experimental group.	No obvious adverse events were observed in every experimental group.
		b. Describe any modifications to the experimental protocols made to reduce adverse events.	To prepare the same volume and same amount of lentiviral particles (TNIP1 shRNA and Con shRNA) before injection will make the protocol more standardized. And higher titer means less volume, which will reduce the skin burden from intradermal injection. -

DISCUSSION			
Interpretation/ scientific implications	18	a. Interpret the results, taking into account the study objectives and hypotheses, current theory and other relevant studies in the literature.	The aim of our study was to verify whether TNIP1 could exaggerate or suppress the imiquimod-induced psoriasis-like dermatitis in mice when downregulated. Our study revealed that mice with decreased TNIP1 were more vulnerable to IMQ-induced psoriasis. These findings are consistent with Callahan's study, in which the authors distinctively knockout TNIP1in dendritic cells.
		b. Comment on the study limitations including any potential sources of bias, any limitations of the animal model, and the imprecision associated with the results[2].	A limitation of this study is the fact that lentivirus infection is quite unspecific in vivo: it not only infects keratinocyte, but also other epidermal cells, including dendritic cells. So the exaggerated IMQ-induced psoriatic dermatitis cannot be only interpreted as a keratinocyte phenomenon.
		c. Describe any implications of your experimental methods or findings for the replacement, refinement or reduction (the 3Rs) of the use of animals in research.	If we could knockout TNIP1 in distinctive cell type, for instance, keratinocyte, the results would be more supportive and convinced. Thus the sacrifice of mice would be more valuable.
Generalisability/ translation	19	Comment on whether, and how, the findings of this study are likely to translate to other species or systems, including any relevance to human biology.	We delivered the lentiviral particles by intradermal injection, and discovered that lenvirus infected not only mice skin but also kidney and spleen. This offered the possibility to study the effects of TNIP1 in systemic lupus erythematosus (SLE), in which kidney was clearly abnormal.
Funding	20	List all funding sources (including grant number) and the role of the funder(s) in the study.	The research leading to these results was funded by the National Science Foundation of China (NO 81101200). The funders had no role in study design, data collection and analysis, decision to publish, or preparation of the manuscript.

References

[1].Lowes, M.A., A.M. Bowcock and J.G. Krueger, Pathogenesis and therapy of psoriasis. Nature, 2007. 445(7130): p. 866-73.

[2].Nair, R.P., et al., Genome-wide scan reveals association of psoriasis with IL-23 and NF-kappaB pathways. Nat Genet, 2009. 41(2): p. 199-204.

[3].Sun, L.D., et al., Association analyses identify six new psoriasis susceptibility loci in the Chinese population. Nat Genet, 2010. 42(11): p. 1005-9.

[4].van der Fits, L., et al., Imiquimod-induced psoriasis-like skin inflammation in mice is mediated via the IL-23/IL-17 axis. J Immunol, 2009. 182(9): p. 5836-45.

[5].Wohn, C., et al., Langerin(neg) conventional dendritic cells produce IL-23 to drive psoriatic plaque formation in mice. Proc Natl Acad Sci U S A, 2013. 110(26): p. 10723-8.
