# Supplementary material for: Downregulation of TNIP1 Expression Leads to Increased Proliferation of Human Keratinocytes and Severer Psoriasis-Like Conditions in an Imiquimod-Induced Mouse Model of Dermatitis
Source: PLoS One. 2015 Jun 5;10(6):e0127957. doi: 10.1371/journal.pone.0127957 (PMC4457880; doi:10.1371/journal.pone.0127957)
Supplement: S1 Table — (DOC) [file pone.0127957.s004.doc]

**Table S1**: Oligonucleotides used for creation of anti-C/EBPβ siRNA

| **siRNA** | **Oligonucleotide sequence** |
| --- | --- |
| siRNA #1 | CGT GGT GTT ATT TAA AGA A |
| siRNA #2 | ACA GCG ACG AGT ACA AGA T |
| siRNA #3 | TGC GCG CTT ACC TCG GCT A |
| nonsilencing siRNA | TTC TCC GAA CGT GTC ACG T |
