# Supplementary material for: Downregulation of TNIP1 Expression Leads to Increased Proliferation of Human Keratinocytes and Severer Psoriasis-Like Conditions in an Imiquimod-Induced Mouse Model of Dermatitis
Source: PLoS One. 2015 Jun 5;10(6):e0127957. doi: 10.1371/journal.pone.0127957 (PMC4457880; doi:10.1371/journal.pone.0127957)
Supplement: S2 Table — (DOC) [file pone.0127957.s005.doc]

**Table S2:** Oligonucleotides used for creation of anti-TNIP1 shRNA expression cassettes

| **shRNA** | **Oligonucleotide sequence** |
| --- | --- |
| shTNIP1 #1 | Sense 5’-ccgg GGA AGA GTC CCA GAT GGA ACT CGA GTT CCA TCT GGG ACT CTT CCT TTT TTg-3’ |
|  | Antisense 5’- aat tca aaa aaG GAA GAG TCC CAG ATG GAA CTC GAG TTC CAT CTG GGA CTC TTC C -3’ |
| shTNIP1 #2 | Sense 5’-ccgg-GCA GAA GCA GCG TGA CTT TCT CGA GAA AGT CAC GCT GCT TCT GCT TTT TTg-3’ |
|  | Antisense 5’-aat tca aaa aaG CAG AAG CAG CGT GAC TTT CTC GAG AAA GTC ACG CTG CTT CTG C-3’ |
| shTNIP1 #3 | Sense 5’-ccgg GCA GAA TGA GTT GCT GAA ACT CGA GTT TCA GCA ACT CAT TCT GCT TTT TTg-3’ |
|  | Antisense 5’-aat tca aaa aaG CAG AAT GAG TTG CTG AAA CTC GAG TTT CAG CAA CTC ATT CTG C-3’ |
| shTNIP1 #4 | Sense 5’-ccgg GAT GAG GAG AAG GCA AGA GAA CTC GAG TTC TCT TGC CTT CTC CTC ATC TTT TTTg-3’ |
|  | Antisense 5’-aat tca aaa aaG ATG AGG AGA AGG CAA GAG AAC TCG AGT TCT CTT GCC TTC TCC TCA TC-3’ |
| negative control | Sense 5’- Ccgg TTC TCC GAA CGT GTC ACG TTT CAA GAG AAC GTG ACA CGT TCG GAG AAT TTT Tg-3’ |
|  | Antisense 5’- aat tca aaa aTT CTC CGA ACG TGT CAC GTT CTC TTG AAA CGT GAC ACG TTC GGA GAA-3’ |
