# Supplementary material for: Downregulation of TNIP1 Expression Leads to Increased Proliferation of Human Keratinocytes and Severer Psoriasis-Like Conditions in an Imiquimod-Induced Mouse Model of Dermatitis
Source: PLoS One. 2015 Jun 5;10(6):e0127957. doi: 10.1371/journal.pone.0127957 (PMC4457880; doi:10.1371/journal.pone.0127957)
Supplement: S3 Table — (DOC) [file pone.0127957.s006.doc]

**Table S3:** Primer sequences used in PCR

| **Targetd gene** | **Sequence of primers** |
| --- | --- |
| shRNA vectors | Forward hU6-F2 5’-TAC GAT ACA AGG CTG TTA GAG AG-3’ |
|  | Reverse pM-R 5’-CTA TTA ATA ACT AAT GCA TGG C-3’ |
| TNIP1-EcoR I | Forward 5’-CTC AAG CTT CGA ATT CGC CAC CAT GGA AGG GAG AGG ACC G-3’ |
|  | Reverse 5’- CCA TGG TGG CGA ATT CCT GAG GCC CCT CAC GGT C-3’ |
| TNIP 1 | Forward 5’- CAG AAT GAG TTG CTG AAA CA -3’ |
|  | Reverse 5’- TCT CCT CAT CTT TGA ATG CT -3’ |
| CK6 | Forward 5’- CTG AAT GGC GAA GGC GTT-3’ |
|  | Reverse 5’-CCA CTG CCG ACA-3’ |
| β-actin | Forward 5’-CAA CCA ACT GGG ACG CCA CT-3’ |
|  | Reverse 5’-GCA CAG CCT GGA TAG CAA C-3’ |
